# Collaboration patterns in the German political science co-authorship network

**DOI:** 10.1371/journal.pone.0174671

**Published:** 2017-04-07

**Authors:** Philip Leifeld, Sandra Wankmüller, Valentin T. Z. Berger, Karin Ingold, Christiane Steiner

**Affiliations:** 1 School of Social and Political Sciences, University of Glasgow, Glasgow, United Kingdom; 2 Geschwister Scholl Institute of Political Science, Ludwig-Maximilians-University (LMU Munich), Munich, Germany; 3 Department of Politics and Public Administration, University of Konstanz, Konstanz, Germany; 4 Institute of Political Science, University of Bern, Bern, Switzerland; 5 Oeschger Center for Climate Change Research, University of Bern, Bern, Switzerland; 6 Department of Environmental Social Sciences, Eawag, Dübendorf, Switzerland; Institut Català de Paleoecologia Humana i Evolució Social (IPHES), SPAIN

## Abstract

Research on social processes in the production of scientific output suggests that the collective research agenda of a discipline is influenced by its structural features, such as “invisible colleges” or “groups of collaborators” as well as academic “stars” that are embedded in, or connect, these research groups. Based on an encompassing dataset that takes into account multiple publication types including journals and chapters in edited volumes, we analyze the complete co-authorship network of all 1,339 researchers in German political science. Through the use of consensus graph clustering techniques and descriptive centrality measures, we identify the ten largest research clusters, their research topics, and the most central researchers who act as bridges and connect these clusters. We also aggregate the findings at the level of research organizations and consider the inter-university co-authorship network. The findings indicate that German political science is structured by multiple overlapping research clusters with a dominance of the subfields of international relations, comparative politics and political sociology. A small set of well-connected universities takes leading roles in these informal research groups.

## Introduction

Political scientists try to describe and explain politics in an objective way. Yet, political science, like any other discipline, is not devoid of social processes that affect the research topics being studied and the results being generated [1,2]. Describing and explaining this social component of political science is therefore important for understanding why we know what we know about politics. In this contribution, we seek to describe the structure of German political science in order to yield a better understanding of the prominent individuals, groups, institutions, and research topics that take the lead in these social processes.

German political science is a rather young discipline. It developed as an autonomous field of research and study only after 1945 [3]. For decades, it was an inward-oriented, non-comparative, and non-cooperative discipline [4]. This has only begun to change in recent years. Nowadays, cooperation of German political scientists within and beyond borders and disciplines becomes “business as usual.” The internationalization or “Americanization” [5] of the national discipline is apparently happening in a more abrupt and radical way than in most other scientific disciplines [6].

The progress within a scientific field and the direction that this progress takes is typically driven by individual “stars” in the field [2] as well as groups of collaborating leading researchers known as “invisible colleges” [1]. A scientific “star” is someone who is strongly visible, highly cited, has a strong track record, and connects multiple other researchers [2]. An “invisible college” is defined as a “network of productive scientists linking separate groups of collaborators within a research area” [1]. Invisible colleges are thus composed of tight and informal connections among highly productive researchers that are leading figures of a “group of collaborators” [1], i.e., a group of interacting researchers that work closely together but have few to no direct interactions with researchers outside their group [1]. Invisible colleges thus link the otherwise separate groups of collaborators [1].

As individual beliefs and ideas tend to be shaped by, or lean towards, congruence with the beliefs and ideas of others with whom the individual interacts [7,8], the scientific practices and theories within a discipline are conditioned by interaction patterns within the social network of a field’s scientists [2,9]. Hence, the analysis of “star” scholars and clusters of collaborating researchers across groups is instrumental for understanding the outputs a discipline ultimately produces. To improve our collective understanding of the social processes guiding political research, we are therefore interested in who the “stars” are, what clusters and groups of collaborating political scientists can be identified, and what research topics they study.

We seek to describe the structure of collaborations in German political science by means of a co-authorship network analysis, where groups of collaborating scientists are identified via graph clustering techniques. By examining the network position of authors, our paper can be classified as author-centric in contrast to a paper-centric approach that utilizes publications as unit of analysis [10,11]. While publication co-authorships are a common way to investigate research collaboration networks, studies also take into account other forms of linkages like citations [12,13], hyperlinks between institutional websites [14] or collaborative research grants [15].

In line with previous research, we acknowledge the important role of co-authorship publications as an academic core activity [16–18]. Co-authorship involves personal and direct communication between scientists and can therefore serve as a proxy for intensive collaboration [19–21] and exchange of ideas, theories, and knowledge [17,18]. Engaging in joint publication activities further intensifies the integration of a discipline and can be seen as essential for scientific progress [18,22]. Co-authorship networks on either the individual, the institutional, or the country level have been investigated in various research contexts such as aquatic vertebrates [23], biodiversity and climate change [24], healthcare interventions [25], psoriasis [26], tropical diseases [27] and tuberculosis [28].

Most analyses of the co-authorship network in German political science draw a rather fragmented picture of the discipline [17,18]. However, this may be an artifact of previous data collection strategies because typically only specific data sources like journals are selected. We therefore not only seek to identify the “stars” and research groups within German political science; we also aim at drawing a complete picture of the domestic discipline in order to evaluate the collaboration network at the individual as well as structural level in an exhaustive way.

To accomplish this task, we coded all publications of the 1,339 researchers in German political science at the postdoctoral or faculty level across 85 university departments or research institutes between 2009 and 2013, resulting in a complex web of scientific collaboration patterns. Based on this set of publications, we created a network where a tie between two researchers was drawn if they engaged in joint publication activities. This permits us to examine the overall topology of the collaboration network but also the role of specific individuals and the manifestation of research clusters in this aggregate structure. We operationalize groups of collaborating researchers as clusters of densely connected actors. Moreover, “stars” are identified via descriptive centrality measures.

Thus a strong added value of the present analysis is that we take into account all types of publications, including articles, book chapters, and monographs. This addresses an important shortcoming in most existing co-authorship network analyses because the transition to a more open and internationally oriented community also entails that some researchers publish predominantly in international journals while others focus on monographs and (contributions to) edited volumes. Focusing on only one type of outlet would likely miss out on important structural aspects.

## Co-authorship networks, invisible colleges, and academic “stars”

The state of a discipline can be described in different ways. Social aspects that matter for the production of scientific findings are related to the degree of internationalization, the existence of distinct subfields, the availability of transparent and predictable career trajectories, the existence of “stars” who influence the field, and their wider webs of colleagues who receive and transport their (and each other’s) ideas. Here, we focus on the domestic structure of the discipline and, more specifically, on the identification of influential individuals and the research clusters they are embedded in.

The theoretical framework that we make use of draws on conceptual distinctions employed by Crane [1]. Crane’s theory of scientific growth posits social networks among a research area’s scientists as its main element: A research area is composed of several “groups of collaborators.” These groups in turn comprise senior researchers, who are typically very productive and define the important research questions of the group, as well as several medium or less productive researchers surrounding the leading scientist. The researchers within a group of collaborators work closely together and form a densely interconnected cluster with few outside ties. Yet, amongst the productive leading scientists of important groups of collaborators, strong informal between-group ties exist. These informal ties enable the leading researchers “to monitor the rapidly changing research ‘front’ and to keep up with new findings” and are essential in connecting the otherwise separate groups of collaborators [1]. This second, informal network between the leading scientists is not easily visible to outside observers because it often spans multiple universities or research institutes and thus cannot be easily recognized by mere formal affiliation. It is therefore described as an “invisible college” [1]. (Note that the definition of an invisible college is highly contested and thus the term “invisible college” can have various meanings [29]. Here, Crane’s definition of the concept is employed.)

In defining an invisible college as network of the leading figures of groups of collaborators, Crane [1] makes use of the concept of “social circles” introduced by Kadushin [30,31] to locate elites in various contexts. Kadushin defines social circles as indirect interaction based on a common interest that is institutionalized to a relatively low degree [30]. Invisible colleges in the sense of Crane hence may also be characterized as social circles that are based on fragmented schools [29].

In Crane’s [1] model of scientific growth, invisible colleges shape the development of a research area in a phase of exponential growth (the phase Kuhn [32] labels “normal science”). Similar to Crane’s investigation in rural sociology [1], also German political science fulfills important pre-conditions for being classified as a research area that is in a phase of exponential growth [18,33]. Previous investigations showed that German political science is a rapidly growing discipline across different research institutes and research areas, has a growing co-authorship rate and an ongoing accrual of new scientists. These factors make political science an ideal case for the study of invisible colleges and the groups of collaborators that the invisible colleges connect [18,33] (see the section on “Previous research on the structure of German political science” below).

Moreover, due to the dynamics of homophily and social influence [7,8], the ideas, theories, and scientific practices across the field are likely to mirror the structure of interactions within the network of German political scientists [2,9]. Thus, identifying densely interconnected clusters of researchers in the co-publication network is an important step in understanding the research output in German political science. For example, the structure of invisible colleges and the research topics of the groups of collaborators, as a result of the underlying growth model of science, may determine the discipline’s focus on some research topics while neglecting others. In this contribution, we therefore analyze in how far the clusters of collaborating researchers in German political science occupy only a subset of the research topics relevant to the discipline at large.

We employ graph clustering techniques to delineate groups of collaborating political scientists. There are some pairs of clusters that are completely unrelated while other pairs of clusters can be considered “neighbors” in the overall network because they are distinct enough to be identified as separate clusters yet share one or two links. An important part in describing the structure of the discipline will be the delineation of such neighboring clusters and the individuals that connect them because these researchers may be able to draw particular visibility from connecting multiple research clusters.

As posited by Moody [2], the connections between core scientists through invisible colleges and the aggregate network structure emerging from these ties “[…] helps explain why core scientists were able to so rapidly diffuse their ideas through the community, and we would expect that those with central positions are likely influential. Newman (2001) turns collaboration itself into a status marker and asks, ‘Who is the Best Connected Scientist?’” [2]. In addition to identifying clusters of collaborating researchers, we therefore analyze who are the best connected and therefore structurally the most influential political scientists in Germany. Moody calls these influential scientists “stars” [2]. We operationalize structural influence by analyzing in how many collaboration steps a researcher can reach everybody else in the collaboration network and how many other pairs of scientists’ shortest collaboration paths a researcher intersects. These theoretical ideas are operationalized by two centrality measures.

Finally, the analysis is extended by aggregating the co-authorship network to the inter-institutional level in order to evaluate if groups of connected universities and research institutes stand out and which research organizations are most central in the German political science co-authorship network. Theoretically, this serves to evaluate more clearly how the structural features of the co-authorship network are related to institutional affiliations.

Therefore, three groups of research questions result from the theoretical frameworks outlined above:

1. What groups of collaborating researchers exist in German political science? What are the research topics predominantly occupied by these research clusters? Do these clusters differ with regard to their preferred publication types?
2. Who are the most central researchers in the German co-authorship network of political science? And are there researchers who float between the clusters and who are thus linked to a significant part of the whole discipline? This question relates to the identification of “bridges” that connect multiple clusters.
3. What is the extent of inter-institutional collaboration in German political science? This question addresses the extent of the informal between-group ties invisible colleges are made up of.

## Previous research on the structure of German political science

A study by Plümper examines the international visibility of different German political science institutes [33]. This is measured by the number of English-language publications in international journals. He reaches the conclusion that in comparison to other European countries, German political scientists rank only moderately in terms of internationalization. Other disciplines like economics and the natural sciences have a higher degree of visibility abroad by comparison [33]. While the number of publications of a department in international journals reached on average 30 per year in the 1990s, already 80 publications per year were produced on average after the year 2000. However, not all political science departments contributed equally to this development; some turned out to be far more internationalized than others (Mannheim and Konstanz as well as Heidelberg and the Wissenschaftszentrum Berlin für Sozialforschung (WZB) in Berlin seem the institutes with the highest international visibility) [33].

Pehl [4] takes a similar approach but investigates articles in selected German academic journals to identify subfields and sub-disciplines within German political science. He finds that Germany was largely detached from international trends for a long time and began to orient itself towards internationalization from the 1990s onwards [4].

Arzheimer and Schoen investigate co-authorship patterns of German political scientists based on data from four selected German political science journals for a period of more than four decades [17]. Compared to the British counterparts, German researchers are found to form looser connections among each other and the German discipline appears to be more fragmented.

While all three studies mentioned above provide an indication of an ongoing internationalization within German political science, the choice of journals considered for their analysis was rather restrictive or selective.

Up to this point, the most far-reaching study on co-authorship networks in German political science has been produced by Metz and Jäckle [18]. Based on 20 representatively chosen German-language journals, they analyzed patterns of cooperation for a time span of 12 years (2000–2011) looking at a total of 5,279 articles. Only 23 percent of these articles under consideration had one or more co-authors. But there seems to be a trend towards increased co-authorship rates over time [18]. Their results further show an asymmetric publication behavior of researchers: A few scholars are responsible for a large share of all publications while some only published a few pieces in the period of investigation. Consequently some researchers in political science have many connections with others and some have very few [18]. This is consistent with the scientific growth models posited by Crane [1]. In a recent publication, Metz and Jäckle [34] turn to a global perspective of political science, examining 67,414 articles that were published in 96 journals between 1990 and 2013. About 40 percent of these articles are written in co-authorship, showing a trend toward enhanced collaboration over time [34]. Most notably, this tendency applies to journals with an empirical (and/or economic) orientation. Furthermore, the authors confirm the described asymmetric publication behavior also in the global context [34].

While Metz and Jäckle lay important groundwork for our analysis, there are two major shortcomings of their data sets we could identify: By focusing only on journal articles, Metz and Jäckle [18,34] are unable to capture the different publication styles of political scientists. The main types of publication for many political scientists are still monographs and book chapters, as the focus on journal publications applies only to a certain part of German political scientists. The second shortcoming is that their data sets consider only one publication language at a time. By analyzing collaboration patterns based on German journals only, the authors completely ignore international publication activities of German scholars [18]. In contrast, non-English publications are not taken into account when the global co-authorship network in political science is examined [34].

Co-authorship networks and bibliographic analyses in political science are prominent also in other countries. Chandra et al. [35] investigate US-American co-authorship structures based on the Social Science Citation Index whereas Cancela et al. [36] examine publication activities in Portuguese political research. Leifeld and Ingold [37] identify subfields and analyze the structure of political science collaboration in Switzerland. With regard to the Swiss case, Bernauer and Gilardi [38] observe differences in the publication behavior of researchers over the course of their career. Furthermore, and in line with the findings of Plümper [33] as outlined above, political science institutes vary strongly in their international visibility. In the neighboring discipline of sociology, co-authorship networks were recently investigated in Romania [39] as well as in Poland, Romania and Slovenia [40].

Despite several attempts to examine publication patterns and the structure of co-authorships in political science and sociology in various settings, all cited studies suffer from the central shortcoming that not all publications and not the full bibliographic history of a researcher are taken into consideration. Most studies systematically underestimate or even completely ignore certain publication types (e.g., monographs and book chapters because they are not listed in electronic citation databases), focus only on specific journals, disregard internationally placed publications, or a combination of these limitations applies. This strongly limits the ability to draw more general conclusions about the structure of social sciences, research clusters, and central researchers. By relying on complete publication records of all national scientists from 1960 onwards, the analysis of the scientific collaboration network in Slovenia forms a notable exception [41,42]. Araújo and colleagues employ a similarly encompassing database of scientific curricula in their analysis of research collaboration in Brazil over the last three decades [43]. We aim to close the existing research gap with regard to German political science.

## The dataset

Data collection proceeded in several steps. First, an exhaustive list of 85 university departments and research institutes hosting political scientists in Germany was created. Second, the websites of each department or institute were browsed, and an encompassing list of affiliated researchers that held a doctorate at the time of data collection was put together. For each of these researchers with a doctoral degree, several attributes like affiliations, gender, seniority status, and web address of the researcher’s personal homepage, publication list, or curriculum vitae (CV) were collected. Third, for each of these researchers, all publications during the years 2009 to 2013 as indicated on each researcher’s CV or homepage were entered into a relational database, along with several variables like type of publication, year, title, co-authors, and editor. The types of publications that were registered comprise journal articles, book chapters, monographs, edited volumes, and “other items,” where the latter category contains working papers, unpublished manuscripts, and book reviews. We did not include newspaper articles.

Previous research indicated that the inclusion of PhD researchers would not add significant structure to the co-authorship network because PhD researchers do not have many publications yet and mainly collaborate with their supervisors, but it would render the data collection task prohibitively more extensive [37].

The main part of the data collection process took place between the fall of 2013 and mid-2014. Researchers from few remaining research institutes were coded at the beginning of 2015. All in all 1,583 researchers were covered. Yet, for 244 political scientists a publicly available publication list or publications that were published during the years 2009 to 2013 could not be identified. These scientists were excluded leaving 1,339 researchers and 22,080 publications for the analysis.

Thus, for each of the 1,339 researchers in the analysis, the dataset includes all publications that each researcher published during the years 2009 to 2013 (i.e., from 1^st^ January of 2009 until the 31^st^ of December 2013). The end date of 2013 was chosen in order not to severely over-represent the publication activity of researchers who update their publication list more frequently and to ensure that for every researcher the time span covered was the same–no matter whether the researcher was coded early or late in the data collection process. The start date of 2009 was chosen to reduce the workload while still yielding a meaningful recent set of publications. Going back further in time would have likely introduced a stronger disadvantage for junior researchers as these persons did not have the same opportunity to publish in earlier years. Any data collection effort necessarily introduces biases in terms of seniority because productivity changes over the career trajectory, hence productivity per year is not easily comparable between researchers with different ranks.

Amongst the researchers not included in the analysis due to missing data, there were slightly more postdocs than professors compared to the overall dataset. Moreover, there was considerable variation across institutes in the availability of publication lists or the presence of publications produced during the time span under study. The spectrum ranges from no missing publication lists and no researchers that have not published during the time span under consideration (e.g., Peace Research Institute Frankfurt (HSFK), University of Frankfurt (Main) or Berlin Social Science Center (WZB)) to nearly 63% of researchers not having a publication list or not having listed a single publication for the years 2009 to 2013 at the Willy Brandt School of Public Policy (Erfurt). A rough inspection of the 244 not included researchers reveals that some of these researchers either did not have an accessible publication list or did not list a single publication for the years 2009 to 2013 because they indeed exhibited no publication activity during these years. This in particular applies to young researchers who had just received a doctorate, older (emeritus) researchers that did not conduct active research anymore, or not (very) active part-time researchers or honorary professors. Data for the 244 non-respondents are partially available through their co-authors, which reduces the missing data problem somewhat. As soon as one researcher listed a publication on his or her CV or homepage, the publication entered the database, and links were established between all co-authors.

Hence, this data collection strategy is as exhaustive as possible, yet it also has some limitations. Most notably, it fails to capture the publication activity of some researchers without accessible publication lists. However, compared to most other co-authorship analyses that miss out on book publications, including chapters in edited volumes, the collection strategy employed here will still draw a more encompassing picture of publication activity. In fact, journal articles comprise only 29.5 percent of the publications in the dataset, and it is well possible that previous research suffers from a severe bias because it focuses only on (a part of) these articles, and the degree of traditionalism versus progressivity along with other properties may be correlated with publishing in books versus international journals. In contrast to what would have been possible with a purely journal-based approach, our dataset can thus be interpreted as being close to a snapshot of publication activity in the years 2009 to 2013 across the universe of political scientists in Germany who hold a doctorate and are affiliated with a German university or research institution. Nevertheless, as our data collection approach fails to capture information that is not disclosed by the scientists, a comparison of journal-based vs. CV-based approaches in capturing the publication activity and co-authorship structure would be a worthwhile investigation for future research.

The process of data collection implies that some publications may be counted multiple times because the publications were listed on the homepages or CVs of multiple co-authors and entered the database several times. However, this does not affect the analysis because it is only the connection that counts in the analysis, not the weight of the connection.

We only report the affiliation of researchers for the time of the data collection; we did not look up more recent affiliations, in order to provide a balanced picture, even if this means that some researchers have moved on to other universities in the meantime.

A rectangular 1,339 x 22,080 matrix was created based on this database, with cell entries of 1 indicating authorship of the column publication by the row actor, and 0 otherwise. This two-mode network matrix was converted into a one-mode network matrix by multiplying the original matrix by its transpose. The resulting square 1,339 x 1,339 one-mode network matrix was weighted, with cell entries indicating the number of publications any two researchers co-authored. The cell entries of this co-authorship matrix were subsequently binarized. For the cluster analysis and for computing centrality scores, a subset of this matrix was retained by removing all researchers who were not connected with the bulk of other researchers via direct or indirect paths. In other words, we conceptualized the one-mode matrix as a network and retained only the giant component while deleting nodes that formed smaller components that were not connected to the rest of the network.

**Fig 1:**
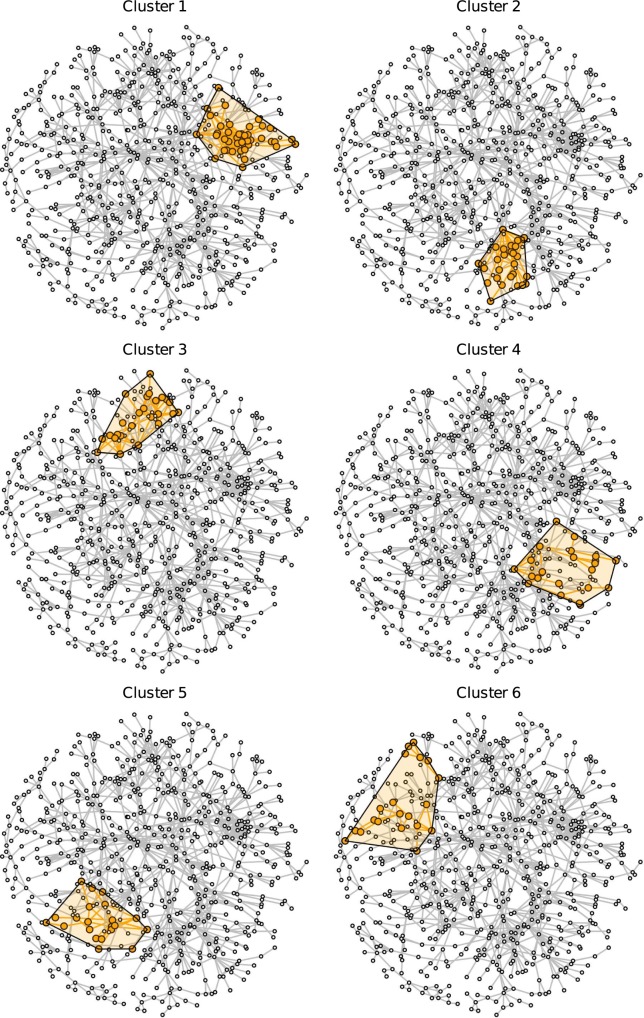
The giant component of the German political science co-authorship network. Orange nodes and shaded polygons denote the largest cohesive subgroups in the network.

**Fig 2:**
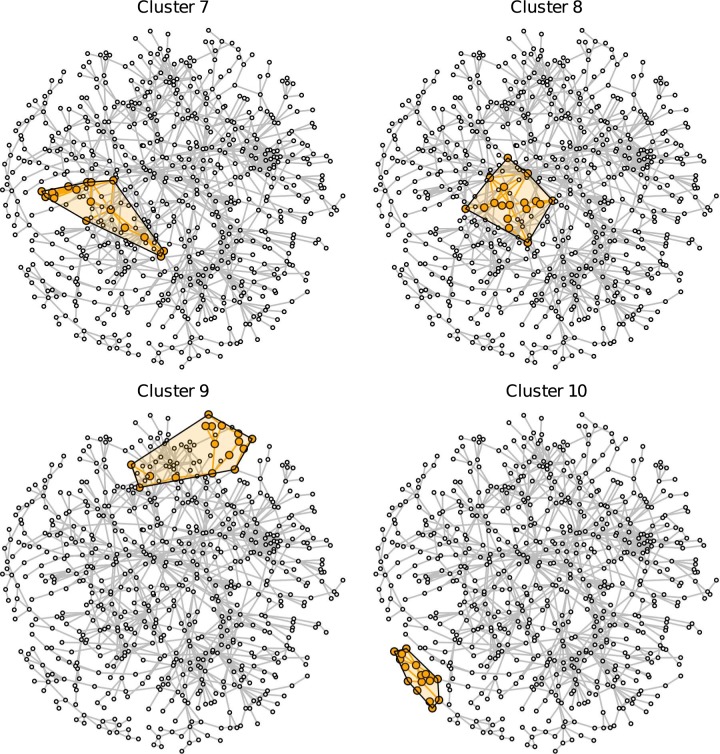
The giant component of the German political science co-authorship network (continued from Fig 1). Orange nodes and shaded polygons denote the largest cohesive subgroups in the network.

The giant component, which can be interpreted as the main political science collaboration network, comprised 673 researchers. Its density (the ratio of the number of realized connections over possible connections) is 0.0052, while the density of the complete network is 0.0015. In other words, the network is sparse compared to many other real-world networks. The giant component network is depicted in Figs 1 and 2 (continued from Fig 1). The diameter of the largest component (i.e., the longest shortest-path distance between any two nodes) is 24 steps. The clustering coefficient (i.e., the tendency of indirectly neighboring nodes to be connected directly) is 0.387, which is relatively high compared to a random graph of the same size and density (which would be about 0.005 for the giant component), mainly because many publications have more than two authors.

## Methodology

The collaboration network serves as a proxy measure for scientific collaboration and the exchange of ideas between researchers. Consequently, if a researcher occupies a central position in the network and connects otherwise disconnected or hard-to-reach parts of the network, this can be interpreted as an indicator of academic influence on the rest of the network. Central nodes are visible to many others, and their input percolates through indirect paths to many other researchers in the network. Therefore we compute the centrality of all researchers in the giant component as a measure of influence in the discipline and list the top 25 researchers below.

There are many centrality measures available. We focus on two measures here for theoretical reasons. Closeness centrality expresses the number of paths a researcher has to traverse in the network in order to reach everybody else in the network, standardized by network size. The theoretical interpretation is that a researcher is close to everybody else and therefore highly visible and influential. Betweenness centrality is another structural centrality measure. It captures the extent to which a researcher occupies the shortest paths between other pairs of researchers. In other words, does the focal researcher connect otherwise distant parts of the network, thereby serving a bridge function between subfields or research topics? (Political science can be partitioned into several broad subfields: *domestic politics* (which sometimes includes political behavior), *comparative politics*, *international relations*, *public policy and administration*, *political theory*, and *methodology*. Our use of the term “research topic” denotes a more fine-grained unit. Any subfield can contain a variety of research topics, and sometimes research topics cut across subfields. For example, research topics could be the *study of welfare states*, the *study of political networks*, or *democratization*.) More details on closeness and betweenness centrality and their formal definitions are provided by Freeman [44].

We are not only interested in the most central individuals, but also in the research clusters present in the collaboration network. What are the distinct and cohesive subgroups of researchers who mutually reinforce each other’s reputation and research agenda? Below, we employ graph clustering techniques in order to identify the ten largest and most distinct groups of collaborating political scientists. Graph clustering, also known as community detection, is a collection of techniques for the identification of cohesive subgroups in networks, i.e., subgraphs with many connections internally but few connections to the outside world [45].

There is a pertinent problem with community detection algorithms: different measures may return slightly different results even though they have the same purpose, and there is no a priori way to determine which solution is “right.” We solve this problem by means of consensus clustering to increase the robustness of the findings. We employ five different, popular graph clustering techniques and aggregate the results into one stable cluster solution. The aggregation rule is that any two researchers have to be grouped into the same cluster by at least four out of five algorithms in order for them to be included in the same cluster. This effectively yields stable core clusters around which other researchers may cluster with lower certainty. In other words, this aggregation produces only the cores of the research clusters for easier interpretation and omits researchers whose affiliation with the respective cluster is ambiguous. The five community detection methods we use are edge-betweenness community detection [45], fast and greedy community detection [46], the Louvain algorithm [47], the Walktrap algorithm [48], and infomap community detection [49]. These methods are among the most widely used community detection algorithms for medium-sized to large networks [50,51] and are readily available in the *igraph* software package [52].

## Results and analysis

In this section, we successively answer the three research questions formulated at the end of the section on “Co-authorship networks, invisible colleges, and academic ‘stars’.” In the first subsection on “Identification of research clusters,” we first plot the locations (i.e., the convex hulls) of the ten largest groups of collaborating researchers returned by the graph consensus clustering approach within the giant component. Then we plot the respective clusters in more detail together with the direct neighborhood in which they are embedded. The location of the nodes in the visualizations is determined by a stress-minimization algorithm (a multidimensional scaling of graph-theoretical distances as implemented in the graph visualization software *visone* [53]). Subsequently, we examine both the giant component and the ten largest research clusters with regard to their composition based on different publication types. In the second subsection on “Researchers with central structural positions in the network,” we analyze the distribution of centrality in the graph and identify the most central researchers. Finally, in the third subsection on the “Aggregation at the level of research organizations,” the co-authorship data are aggregated and analyzed as a network of weighted inter-institutional links between research organizations.

### Identification of research clusters

Figs 1 and 2 (continued from Fig 1) display the locations of the ten largest groups of collaborating researchers within the collaboration network. The core members of the clusters and their mutual connections are colored in orange while all other researchers are colored in white. A shaded polygon denotes the convex hull of these core members, i.e., the minimal area occupied by the nodes identified as core members of the research cluster. The clusters cover all densely populated subgroups in the network.

**Fig 3:**
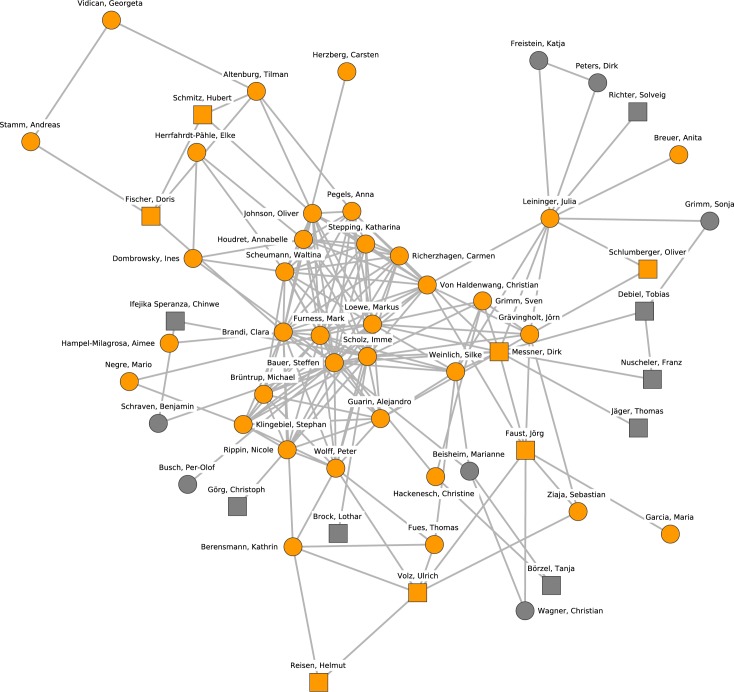
Cluster 1. Cluster 1 (orange nodes) and its direct neighborhood (gray nodes). Cluster 1 is the largest cohesive subgroup identified in the network. Nodes with rectangular shape denote professors. Circles denote postdoctoral researchers.

Fig 3 shows the largest research group (cluster 1) in more detail. Cluster members are denoted by orange nodes, and their direct neighborhood is plotted as gray nodes. Square-shaped nodes denote professors, and circles denote postdoctoral or other non-professorial research staff. The vast majority of the 42 core researchers are current members of the German Development Institute (DIE). There are frequent collaborations going on within the DIE compared to the rest of political science in Germany. The cluster has a densely interconnected core that consists of (non-professorial) researchers such as Markus Loewe, Mark Furness, Steffen Bauer and Imme Scholz. The research topics in this cluster are related to the research focus of the DIE such as development, political economy in developing countries, international collaboration, area studies, and sustainability. Cluster 1 has sparse connections to other political scientists. However, the cluster does maintain collaborations with prominent researchers in international relations, e.g., Tanja Börzel and Marianne Beisheim.

**Fig 4:**
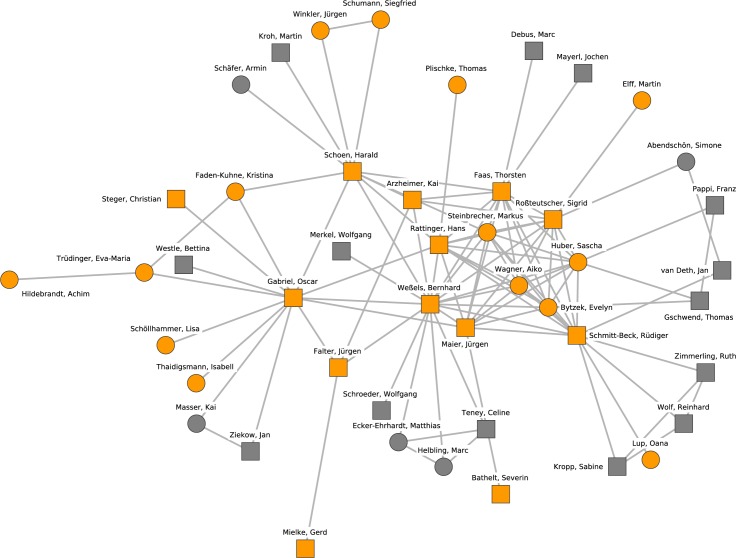
Cluster 2. Cluster 2 (orange nodes) and its direct neighborhood (gray nodes). Cluster 2 is the second-largest cohesive subgroup identified in the network. Nodes with rectangular shape denote professors. Circles denote postdoctoral researchers.

Fig 4 shows details on the second-largest cluster. The researchers in this cluster are united by their joint interest in elections and voting, political sociology, and German politics. The center of this cluster is composed of the “Mannheim School,” a number of quantitative researchers with current affiliations, former training at, or close connections with, the University of Mannheim, with a focus on the quantitative study of voting and elections. Besides the University of Mannheim, prominent members are affiliated to the University of Mainz (Kai Arzheimer, Thorsten Faas, Jürgen Falter) and the University of Stuttgart (Oscar Gabriel). Core members of this research cluster are linked due to their collaboration in the course of the German Longitudinal Election Study (GLES), either as principal investigators (Hans Rattinger, Sigrid Roßteutscher, Rüdiger Schmitt-Beck, Harald Schoen, Bernhard Weßels) or as current or former research fellows (Evelyn Bytzek, Sascha Huber, Markus Steinbrecher, Aiko Wagner).

**Fig 5:**
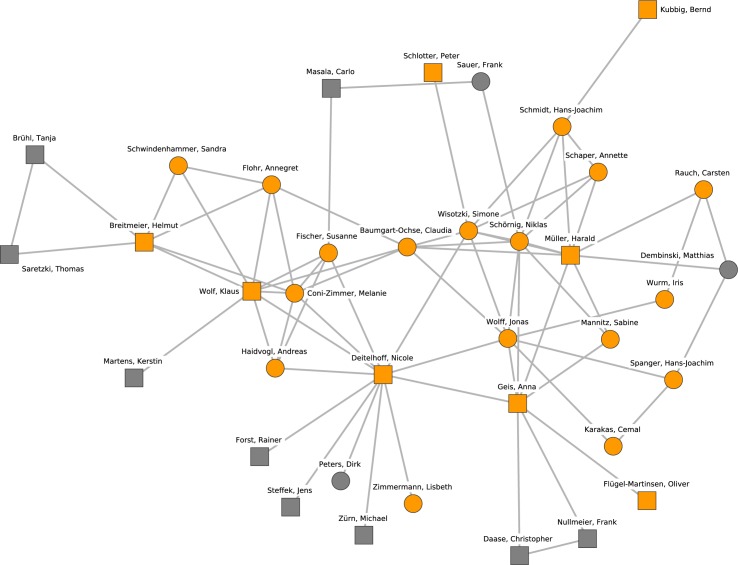
Cluster 3. Cluster 3 (orange nodes) and its direct neighborhood (gray nodes). Cluster 3 is the third-largest cohesive subgroup identified in the network. Nodes with rectangular shape denote professors. Circles denote postdoctoral researchers.

Fig 5 shows a cluster from the subfield of international relations with democratization, armament, conflict, and peace research as the most prominent research topics in this research group. Cluster 3 mostly consists of professors and postdoctoral researchers from either the Peace Research Institute Frankfurt (HSFK) or the University of Frankfurt (Main) as well as other research institutions from the Rhine-Main Area.

**Fig 6:**
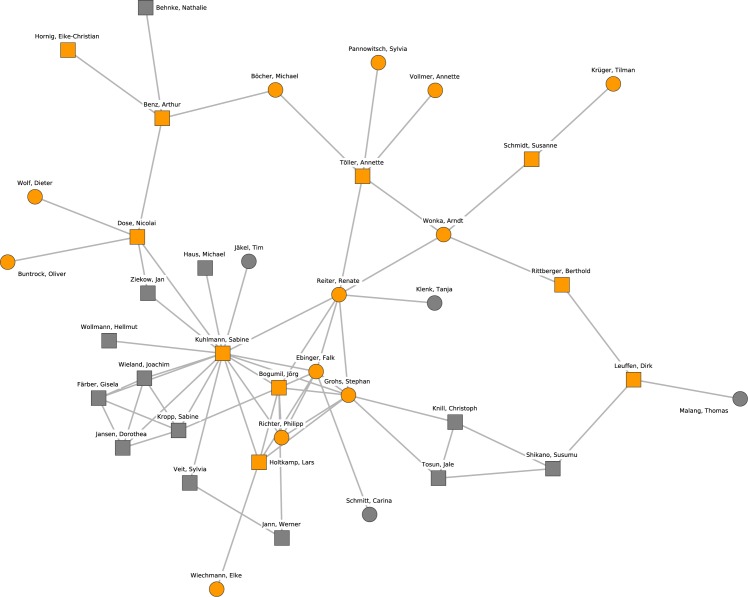
Cluster 4. Cluster 4 (orange nodes) and its direct neighborhood (gray nodes). Cluster 4 is the fourth-largest cohesive subgroup identified in the network. Nodes with rectangular shape denote professors. Circles denote postdoctoral researchers.

Research cluster 4 presented in Fig 6 is predominantly concerned with public administration, public sector reform, and organization studies. The central nodes in this community are affiliated with the universities in Bochum (Jörg Bogumil), Hagen (Lars Holtkamp, Renate Reiter), Konstanz (Falk Ebinger, Stephan Grohs), Potsdam (Philipp Richter) and Speyer (Sabine Kuhlmann). Furthermore, a web of adjacent nodes formed by professors of the German University of Public Administration in Speyer (Gisela Färber, Dorothea Jansen, Sabine Kropp and Joachim Wieland) that are in the direct neighborhood of this cluster and linked to Sabine Kuhlmann attracts attention.

**Fig 7:**
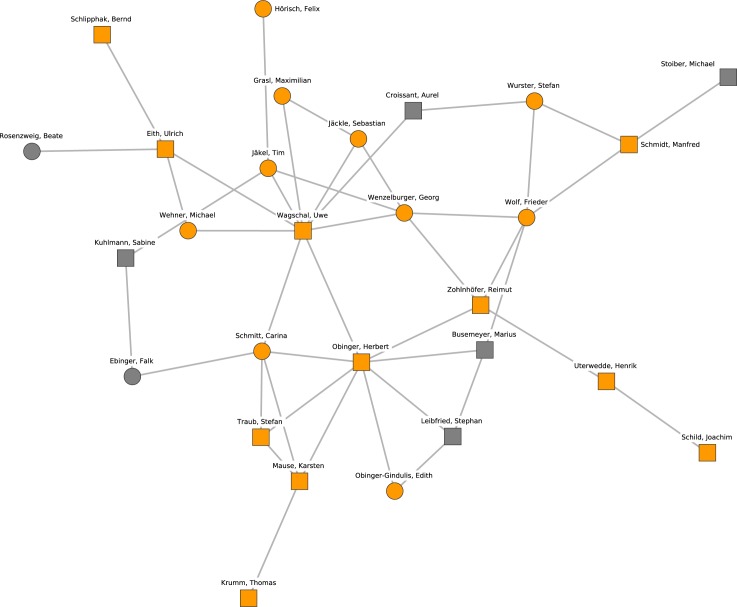
Cluster 5. Cluster 5 (orange nodes) and its direct neighborhood (gray nodes). Cluster 5 is the fifth-largest cohesive subgroup identified in the network. Nodes with rectangular shape denote professors. Circles denote postdoctoral researchers.

Fig 7 shows cluster 5, which revolves around the research topics of social policy, the welfare state, political economy, and comparative politics. The central scientists of this research cluster are Uwe Wagschal and Georg Wenzelburger (Freiburg), Herbert Obinger and Carina Schmitt (Bremen) as well as Reimut Zohlnhöfer and Frieder Wolf (Heidelberg).

**Fig 8:**
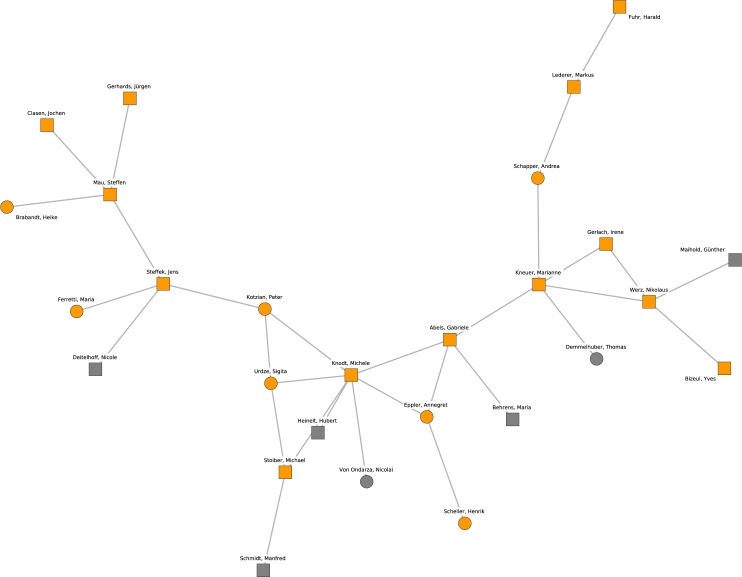
Cluster 6. Cluster 6 (orange nodes) and its direct neighborhood (gray nodes). Cluster 6 is the sixth-largest cohesive subgroup identified in the network. Nodes with rectangular shape denote professors. Circles denote postdoctoral researchers.

Cluster 6, which is depicted in Fig 8, originates from the subfield of comparative politics, especially the comparison of political systems with a focus on the European Union. Prominent members of this cluster are the professors Michele Knodt (Darmstadt), Gabriele Abels (Tübingen) and Marianne Kneuer (Hildesheim) that link scientists from various universities. This comparative politics cluster is adjacent to the previous cluster 6 with its similar focus on comparative and welfare state politics (see connections through Michael Stoiber and Manfred G. Schmidt).

**Fig 9:**
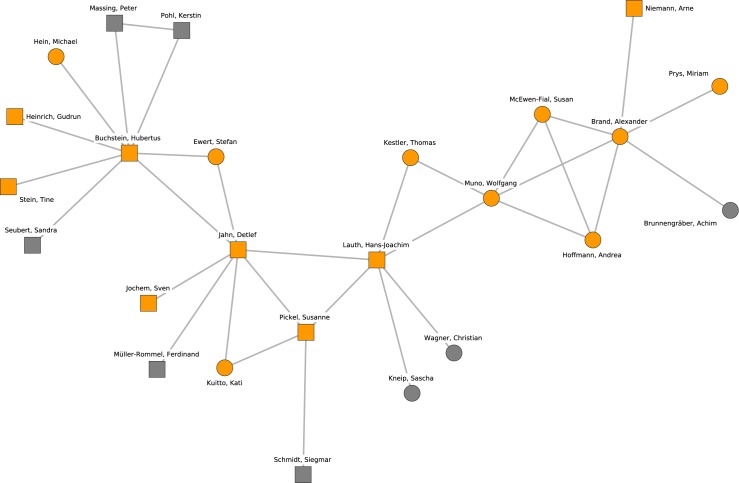
Cluster 7. Cluster 7 (orange nodes) and its direct neighborhood (gray nodes). Cluster 7 is the seventh-largest cohesive subgroup identified in the network. Nodes with rectangular shape denote professors. Circles denote postdoctoral researchers.

Fig 9 reveals that the center of cluster 7 also developed within the subfield of comparative politics. Central members of this cluster are linked by publishing a textbook on the methods of comparative social sciences, being either an editor (Detlef Jahn, Hans-Joachim Lauth, Susanne Pickel) or a contributor (Wolfgang Muno). In general, cluster 7 is a highly diverse cluster that connects researchers with an interest in political theory (upper left) to researchers with a regional focus on Latin America (right).

**Fig 10:**
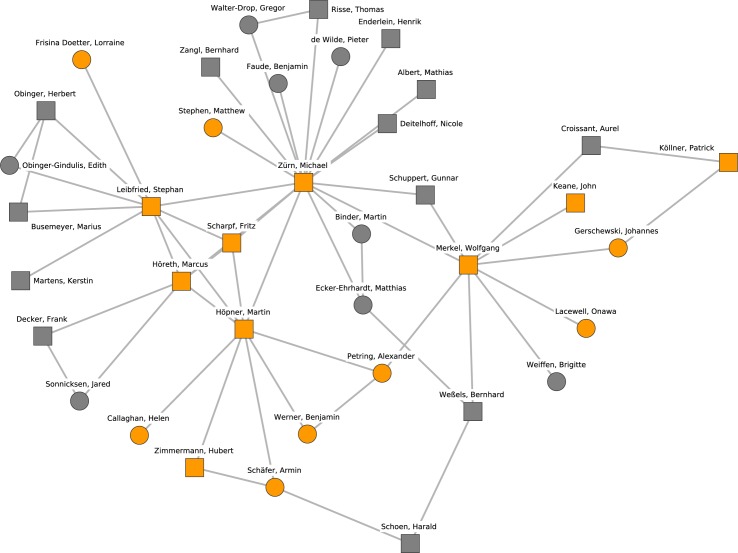
Cluster 8. Cluster 8 (orange nodes) and its direct neighborhood (gray nodes). Cluster 8 is the eight-largest cohesive subgroup identified in the network. Nodes with rectangular shape denote professors. Circles denote postdoctoral researchers.

Cluster 8, which is presented in Fig 10, can be characterized as being composed of researchers that examine the transformation of states from a global governance, democratization or political economy perspective. The Berlin Social Science Center (WZB) with Michael Zürn and Wolfgang Merkel as well as the Max Planck Institute for the Study of Societies based in Cologne with Martin Höpner and Fritz Scharpf are central for this research cluster. Further prominent researchers are Marcus Höreth (Kaiserslautern) and Stephan Leibfried (Bremen). Remarkably, Michael Zürn connects the cluster with eight adjacent nodes, some of whom are members of other clusters (more details below). Cluster 8 is adjacent to cluster 5 with its focus on welfare states and comparative politics; both clusters focus on political institutions.

**Fig 11:**
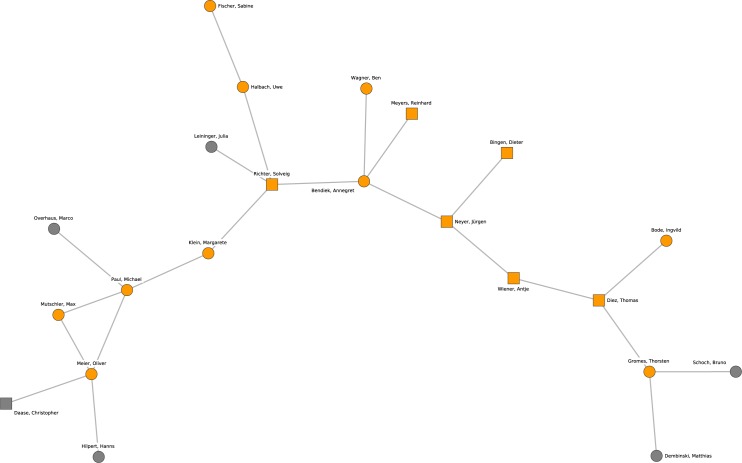
Cluster 9. Cluster 9 (orange nodes) and its direct neighborhood (gray nodes). Cluster 9 is one of the ninth-largest cohesive subgroups identified in the network. Nodes with rectangular shape denote professors. Circles denote postdoctoral researchers.

Fig 11 shows research cluster 9 that is based on the subfield international relations and puts emphasis on theory especially in the context of the European Union and European integration. Cluster 9 connects professors from Tübingen (Thomas Diez), Hamburg (Antje Wiener) and Frankfurt (Oder) (Jürgen Neyer) with researchers sharing a background from the German Institute for International and Security Studies (SWP).

**Fig 12:**
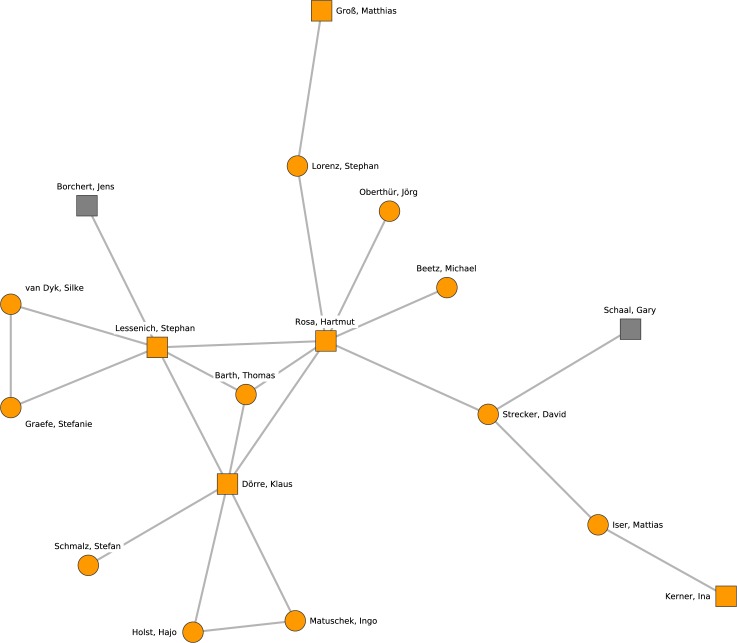
Cluster 10. Cluster 10 (orange nodes) and its direct neighborhood (gray nodes). Cluster 10 is the one of the ninth-largest cohesive subgroups identified in the network. Nodes with rectangular shape denote professors. Circles denote postdoctoral researchers.

The last research cluster presented here is depicted in Fig 12. Cluster 10 is located at the borderline between political science and sociology and its research takes a critical perspective on society and global capitalism. The majority of professors (Hartmut Rosa, Stephan Lessenich, Klaus Dörre) and postdoctoral researchers are affiliated with the University of Jena.

This description of the 10 largest research clusters exhibits a number of interesting features. Most notably, political science in Germany appears to be a diversified discipline but is much stronger in international and comparative politics than in other subfields like public policy, domestic politics, or methodology, which are stronger in neighboring countries like Switzerland [37] and the Netherlands. All in all, four clusters are related to the subfield of international relations, three clusters cover research topics of comparative politics, two clusters address political sociology and one cluster focuses on public administration.

Second, Crane [1] characterizes invisible colleges as multiple groups of collaborators with cross-institutional (informal) collaboration ties and a star-like connection from more towards less senior researchers. These structural features are mostly observable for the clusters 2, 3, 4, 5, 8, and 10, but less so for clusters 1, 6, 7, and 9. The latter can, however, still be considered as groups of collaborators mainly interested in one or two specific topics.

Third, there are substantial collaborations between the clusters addressing a subfield, but also between the clusters with a diverse focus (e.g., clusters 4, 5, and 8 are adjacent and have similar research topics, but different sub-foci, such as welfare states as opposed to democratization in comparative politics).

Fourth, the identified groups of collaborating researchers are mainly defined by institutional connections (e.g., collaborations within DIE, University of Frankfurt (Main) and HSFK, or Jena).

Fifth, there are examples of research clusters where universities with similar approaches, rather than research topics, have frequent exchanges of staff and thereby collaborations (Mannheim, Mainz and Stuttgart).

Sixth, the discipline within Germany is relatively well-connected, with a diameter of 24 in the giant component despite the fact that there are 673 members in this component.

**Fig 13:**
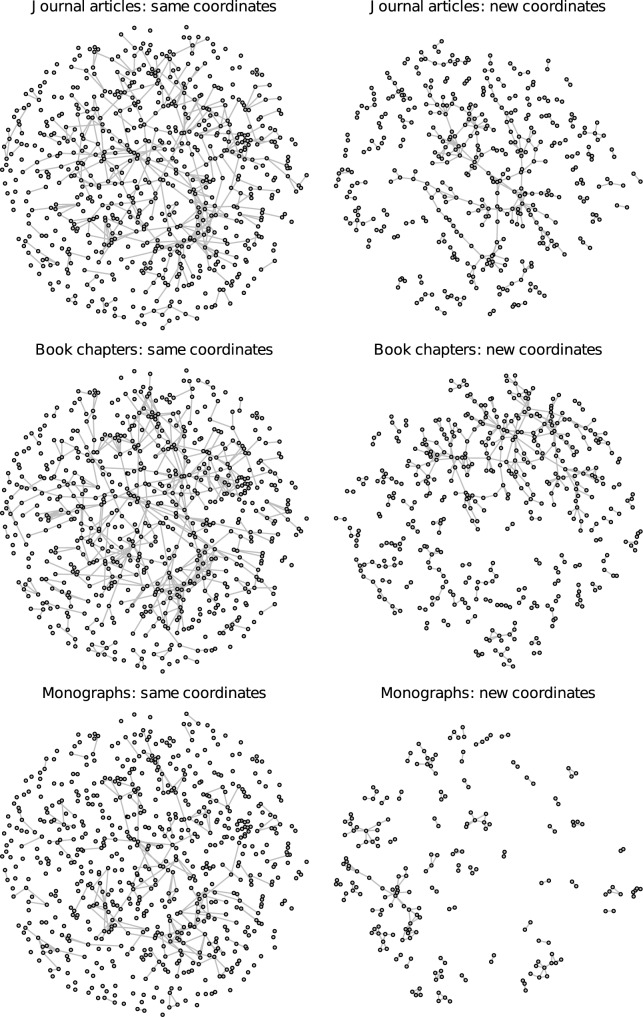
Co-authorships by publication type. Connections by publication type using the same coordinates as in Figs 1 and 2 (on the left) and a new layout without isolates (on the right). Journal articles and book chapters account for most connections.

**Fig 14:**
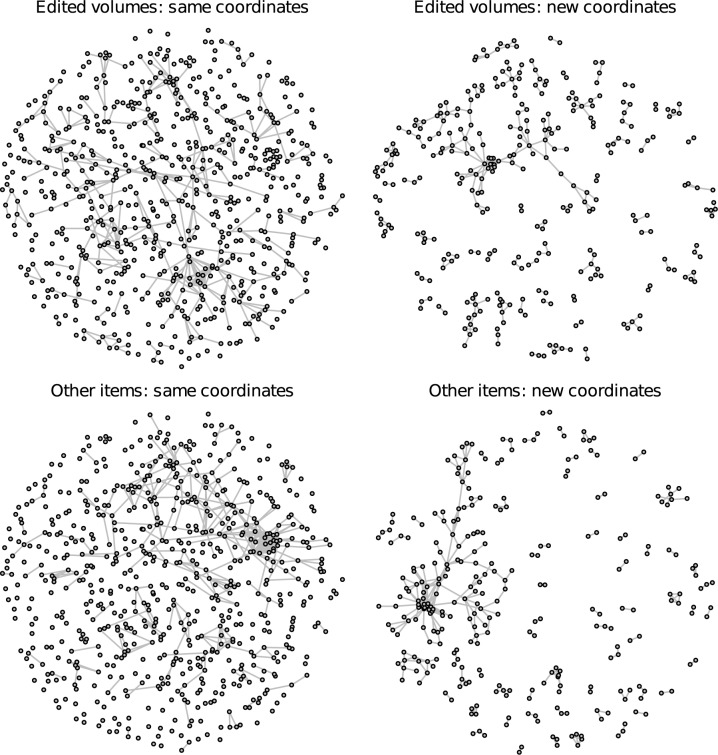
Co-authorships by publication type (continued from Fig 13). Connections by publication type using the same coordinates as in Figs 1 and 2 (on the left) and a new layout without isolates (on the right). Journal articles and book chapters account for most connections.

Hence, geographical proximity as well as assortativity (i.e., homophily and/or social influence) with regard to thematic or scientific approaches seem to shape the co-authorship network of German political scientists and the clusters that emerge within this network. Another aspect that, due to the forces of homophily and social influence, is likely to vary across the identified clusters are the preferred publication types. To examine the distribution of different publication types over the giant component and the identified clusters, Figs 13 and 14 (continued from Fig 13) first show how the network is composed of different publication types. The left column displays the network with the same node coordinates as in Figs 1 and 2. A comparison permits an evaluation of which regions of the network focus particularly on a specific kind of publication activity. The right column visualizes the same data but rearranges the nodes using a standard graph layout and excludes isolates in order to give a clearer visual impression of the topology of the subgraph induced by each publication type. Journal articles, book chapters, and edited volumes span different regions of the network while monographs and other, unpublished items (mainly working papers and unreleased manuscripts and a few book reviews; no newspaper articles were included in the data collection) are concentrated more locally in certain research clusters, which is partly due to their lower numbers. The residual category of other items is mainly concentrated in clusters 1 and 3.

**Fig 15:**
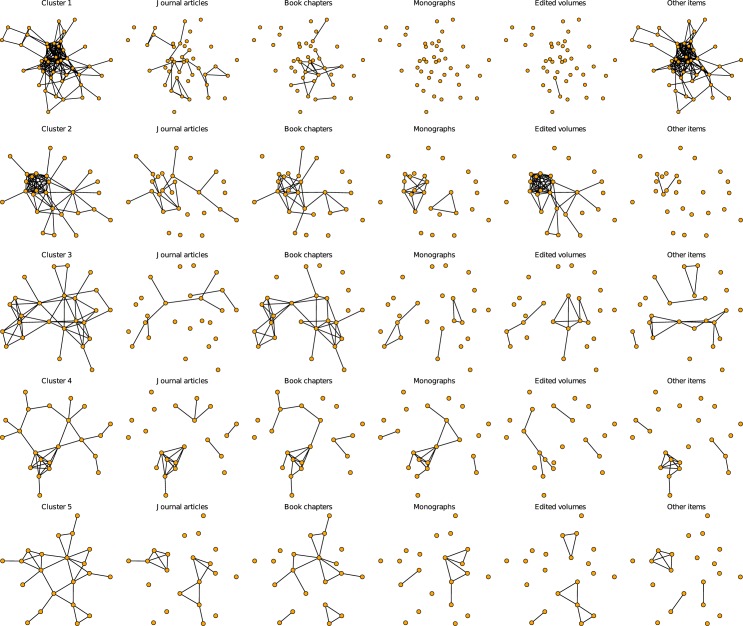
Clusters and their composition by publication type. Orange nodes denote members of the clusters from Figs 1 and 2. The first column shows the complete cluster; the remaining columns show the subset of edges formed through collaborations on journal articles (second column), book chapters (third column), monographs (fourth column), edited volumes (fifth column), and other items (sixth column).

**Fig 16:**
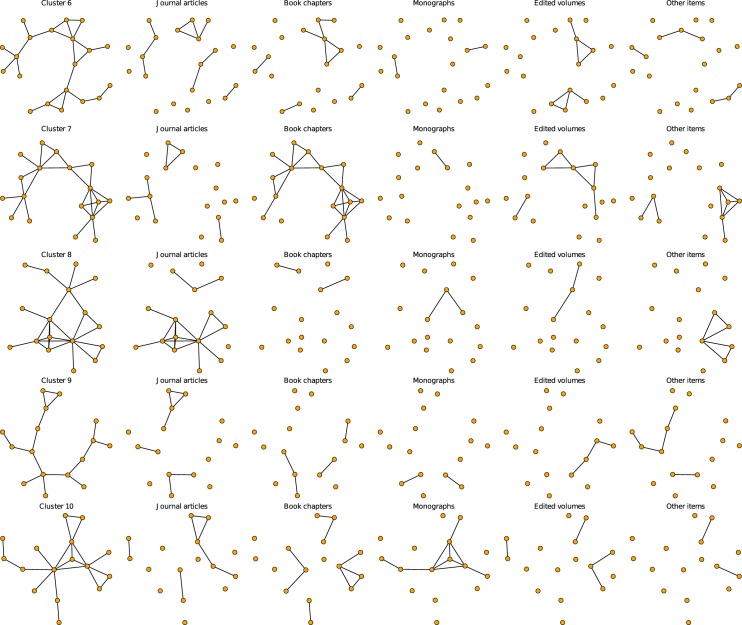
Clusters and their composition by publication type (continued from Fig 15). Clusters and their composition by publication type. Orange nodes denote members of the clusters from Figs 1 and 2. The first column shows the complete cluster; the remaining columns show the subset of edges formed through collaborations on journal articles (second column), book chapters (third column), monographs (fourth column), edited volumes (fifth column), and other items (sixth column).

To evaluate the composition of clusters by publication type more clearly, Figs 15 and 16 (continued from Fig 15) visualize the ten clusters for each publication type separately. Orange nodes are cluster members, and gray nodes are their adjacent nodes. “Other items” is the modal category in cluster 1. The distributions also show that non-journal publications such as books and book chapters are an important component of the network. Disregarding these publications, which is the common practice in many co-authorship analyses, may alter the topology significantly. We deliberately chose this very inclusive data collection strategy as we are interested in the social dimension of collaboration patterns and their resulting topological features and subgroups, irrespective of the scientific quality of the collaborations (as indicated by different publication types). Joint journal publications are the modal category only in clusters 6 and 8. Book chapters and edited volumes are the modal joint publication type in cluster 2, 3, 4, 5, 7 and 10.

### Researchers with central structural positions in the network

Who are the most central researchers in the German political science collaboration network? We focus on the giant component and compute two centrality indices: closeness centrality and betweenness centrality, shown in Tables 1 and 2.

**Table 1:** Top 25 betweenness-central political scientists in Germany and their affiliations.

Name	Betweenness	Affiliation
Zürn, Michael	0.0339	FU Berlin & WZB
Weßels, Bernhard	0.0285	HU Berlin & WZB
Beisheim, Marianne	0.0249	FU Berlin & SWP
Merkel, Wolfgang	0.0231	HU Berlin
Gabriel, Oscar	0.0201	FÖV Speyer & Stuttgart
Kuhlmann, Sabine	0.0194	FÖV Speyer & Potsdam
Wagner, Christian	0.0158	SWP
Börzel, Tanja	0.0155	FU Berlin
Deitelhoff, Nicole	0.0148	Frankfurt
Zangl, Bernhard	0.0144	LMU Munich
Westle, Bettina	0.0139	Marburg
Croissant, Aurel	0.0138	Heidelberg
Abendschön, Simone	0.0129	Frankfurt
Reiser, Marion	0.0125	Frankfurt & Hamburg
Roßteutscher, Sigrid	0.0123	Frankfurt
Borchert, Jens	0.0121	Frankfurt
Lessenich, Stephan	0.0117	Jena
Kropp, Sabine	0.0115	FU Berlin & FÖV Speyer
Bauer, Steffen	0.0113	DIE
Ziekow, Jan	0.0108	Speyer
Wagschal, Uwe	0.0099	Freiburg & Willy Brandt School of Public Policy
Lauth, Hans-Joachim	0.0092	Würzburg
Brock, Lothar	0.0089	HSFK
Knill, Christoph	0.0089	Konstanz
Scholz, Imme	0.0089	DIE

**Table 2:** Top 25 closeness-central political scientists in Germany and their affiliations.

Name	Closeness	Affiliation
Zürn, Michael	0.2008	FU Berlin & WZB
Merkel, Wolfgang	0.1968	HU Berlin
Zangl, Bernhard	0.1921	LMU München
Beisheim, Marianne	0.1896	FU Berlin & SWP
Börzel, Tanja	0.189	FU Berlin
Weßels, Bernhard	0.1875	HU Berlin & WZB
Risse, Thomas	0.1869	FU Berlin
Ecker-Ehrhardt, Matthias	0.1851	FU Berlin
Deitelhoff, Nicole	0.1819	Frankfurt
Croissant, Aurel	0.1818	Heidelberg
Schuppert, Gunnar	0.1793	FU Berlin & Erfurt
Binder, Martin	0.1792	WZB
Albert, Mathias	0.1789	Bielefeld
Brock, Lothar	0.1786	HSFK
Leibfried, Stephan	0.1786	Bremen & BIGSSS
Knill, Christoph	0.1784	Konstanz
Genschel, Philipp	0.1777	BIGSSS
Buzogany, Aron	0.1764	LMU Munich & FU Berlin & FÖV Speyer
Höpner, Martin	0.1761	Köln
Kropp, Sabine	0.1758	FU Berlin & FÖV Speyer
Kuhlmann, Sabine	0.1757	Potsdam & FÖV Speyer
Helbling, Marc	0.1751	WZB
Bauer, Michael	0.175	Speyer
Bauer, Steffen	0.175	DIE
Weiffen, Brigitte	0.1749	Tübingen & Konstanz

Betweenness centrality of a researcher is proportional to the number of shortest paths between any two other researchers the focal researcher is situated on. This measure therefore captures the extent to which a given scientist connects different parts of the network, or different groups of collaborating scientists. Hence it is a complementary measure to the identification of clusters because it serves to identify the “bridges” between these clusters and between other researchers within the collaboration network. Nodes with a high betweenness centrality score are influential since they are able to reach out to otherwise relatively distant parts of the network. Thereby, they can control the percolation of others’ influence across research clusters, and they can in turn influence these other clusters.

**Fig 17:**
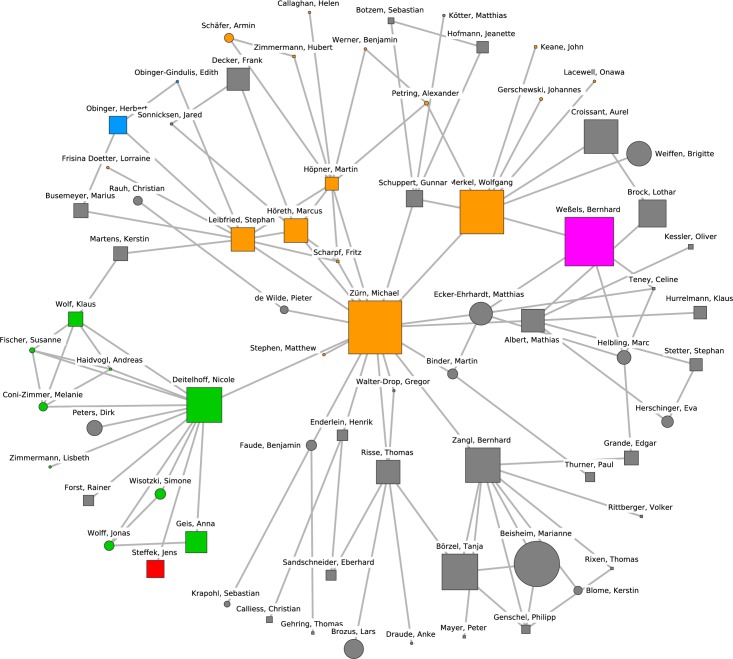
First- and second-order ego network of the most central German political scientist. The most central German political scientist, Michael Zürn (WZB & FU Berlin), and his first- and second-order ego network. Orange nodes are political scientists in cluster 8. Green nodes belong to cluster 3. Red nodes are part of cluster 6. Blue nodes belong to cluster 5. Pink nodes are part of cluster 2. Gray nodes are not part of any of the ten largest clusters presented here. These gray nodes nevertheless may belong to the direct neighborhood of other clusters. Tanja Börzel and Marianne Beisheim, for example, are part of the direct neighborhood of cluster 1. The size of the nodes is proportional to a researcher’s betweenness centrality. Squares denote professors; circles denote postdoctoral researchers or non-professorial academic staff with a doctoral degree.

The most betweenness-central node is Michael Zürn (FU Berlin and WZB). To understand how Michael Zürn could become the center of the German political science universe, one needs to consider the different groups he is connecting. Fig 17 shows his first-order and second-order ego network, that is, his neighbors and their neighbors in the network. Michael Zürn, himself being part of cluster 8, directly or indirectly connects important members of cluster 3 (highlighted in green; e.g. Nicole Deitelhoff, Klaus Wolf, Anna Geis), cluster 6 (Jens Steffek, highlighted in red), cluster 5 (Herbert Obinger, highlighted in blue), and cluster 2 (Bernhard Weßels, highlighted in pink). Moreover, Michael Zürn is indirectly linked to Tanja Börzel and Marianne Beisheim who are in turn in the direct neighborhood of cluster 1. Hence Michael Zürn has an immense connective capacity and–insofar as betweenness centrality in the co-authorship network can be assumed to translate into scientific influence–is likely to have a high influence on various research clusters in German political science across distinct research topics represented by the different clusters.

**Fig 18:**
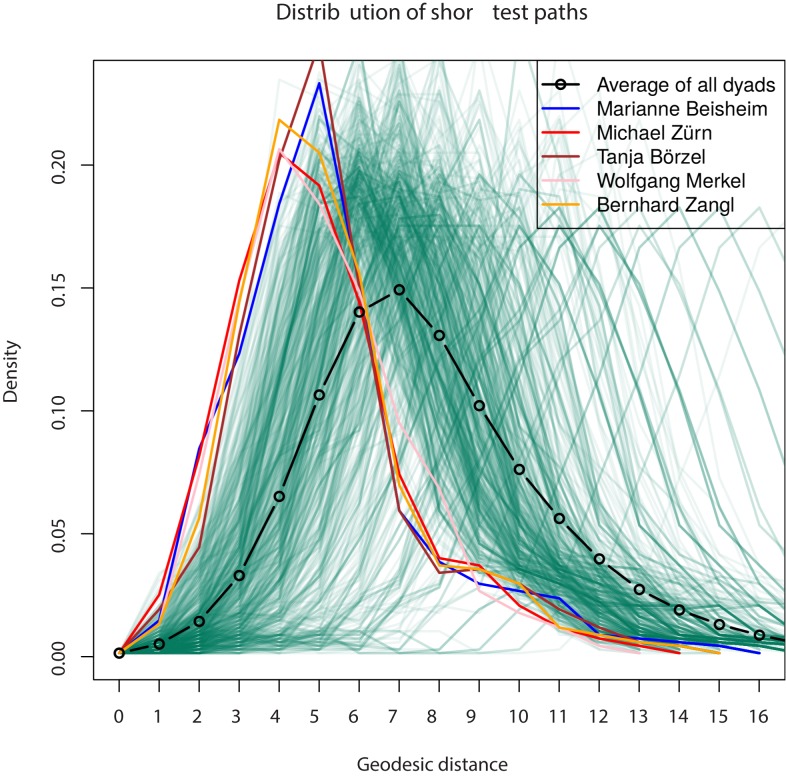
Distribution of shortest path lengths. Distribution of shortest path lengths between a focal researcher and all remaining nodes of the giant component. Average density and selected densities for the top five closeness-central researchers are highlighted.

Closeness centrality (Table 2) captures how many steps removed from all other nodes a researcher is. This is an indication of a researcher’s potential outreach and influence on other researchers. As in the previous case, Michael Zürn is the most centrally connected researcher. Fig 18 shows the distribution of path lengths to all other researchers from any given researcher, with the top five closeness-central nodes highlighted. Michael Zürn (cluster 8), Wolfgang Merkel (cluster 8), Bernhard Zangl, Marianne Beisheim and Tanja Börzel have the shortest average path lengths to any other researcher in the network—a value of about 5 steps, while the average value in the dataset is approximately 7 steps. Thus it is fair to speak of “seven degrees of separation” [19] in the German political science collaboration network.

To conclude, Michael Zürn as well as other highly central researchers like Wolfgang Merkel, Bernhard Zangl, Marianne Beisheim and Tanja Börzel or Bernhard Weßels seem to be the visible and potentially highly influential academic “stars” of German political science.

### Aggregation at the level of research organizations

A share of 33 percent of all ties in the disaggregated co-authorship network occurs within institutions, and 67 percent of collaboration ties occur across research organizations. We aggregated the dataset and created a weighted network indicating collaboration strength between the 85 research organizations in the dataset. To assess each university’s or research institute’s tendency to have inbound, rather than outward, connection profiles, we computed the EI-index [54], which is bound between 0 (for purely inward collaboration profiles) and 1 (for purely outbound collaboration profiles). The results are not reported here in full as most research organizations have an overwhelmingly outward-bound collaboration profile. There are, however, some noteworthy cases with significant internal collaboration. The DIE institute has an EI value of 0.80, followed by FU Berlin (0.90), the SWP (0.94), GIGA Hamburg (0.95), the University of Freiburg (0.95), and the University of Konstanz (0.96), with only marginal differences between all remaining institutions.

Besides affiliation, the effect of geographic distance on the tendency to collaborate was tested in a logistic regression analysis with a permutation test using the quadratic assignment procedure (QAP) [55,56]. The logarithm of the geographic distance (measured in kilometers) had a statistically discernible effect on co-authorship at the 95 percent significance level (β = -0.26; Pr(≤b) = 0.0002; Pr(≥|b|) = 0.0002; 5,000 permutations).

**Fig 19:**
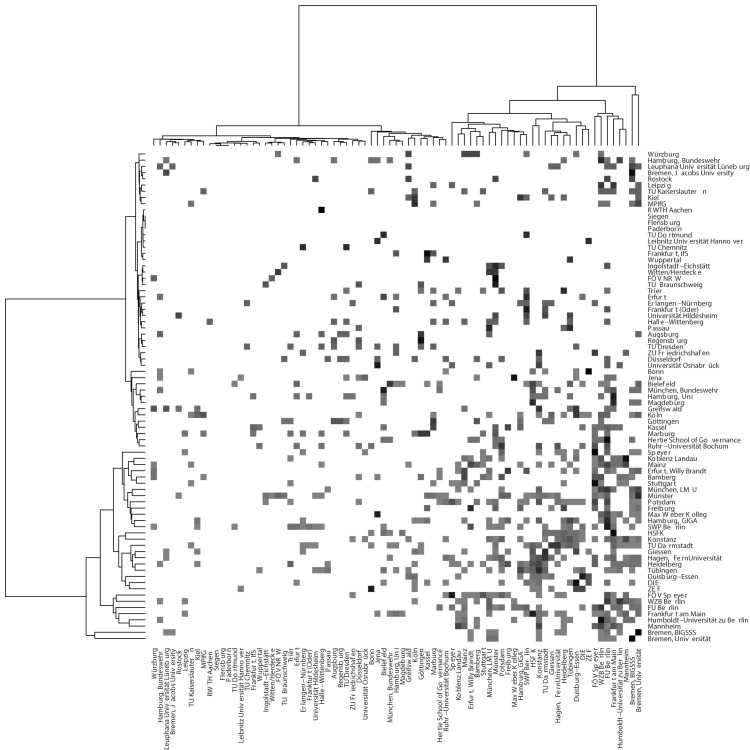
Heat map of the co-authorship network aggregated at the level of research organizations. Weights in the network denote how many individual collaboration ties between researchers exist across any two universities or research institutes. The dendrograms show the result of a hierarchical cluster analysis based on Ward’s criterion (with non-squared distances).

**Fig 20:**
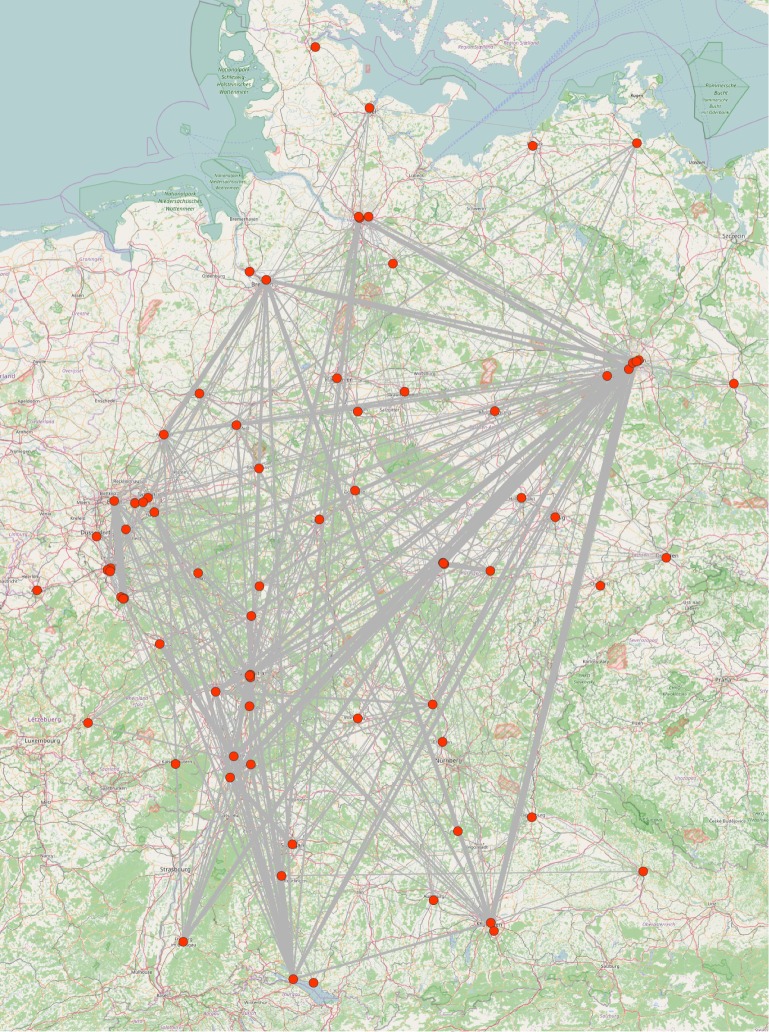
Inter-institutional co-authorship network projected on a map of Germany. Aggregated network between research organizations with geographical coordinates plotted on a map using the software *visone*.

Figs 19 and 20 show the network aggregated at the university level. Fig 19 is a heat map with a hierarchical cluster analysis and shows that an inner circle of research organizations has a relatively strong tendency to collaborate with each other (lower right quarter) while the remaining organizations tend to publish with each other (upper left quarter) and with those organizations in the lower right quarter (off-diagonal blocks) only to a minor degree. Geographically proximate institutions collaborate in particular, and partly this is due to institutional linkages (or vice-versa). Fig 20 projects the inter-institutional co-authorship network on a map of Germany. Regional collaboration clusters such as among organizations in Berlin, the Ruhr valley, or northern Baden-Württemberg and the Rhine Mine Area are visible. The universities and research institutes in Berlin are particularly involved in external research activities. This underlines the central role of researchers from institutions in Berlin in the disaggregated co-authorship network identified above.

## Concluding remarks

In this contribution, we identified the cores of the ten most distinct research clusters in German political science. We also highlighted the most central researchers in this collaboration network, who are likely able to exert influence by connecting otherwise disconnected clusters. Finally, we analyzed the role of geographic location in the co-authorship network. Besides many specific findings, two more general patterns deserve particular attention:

First, in contrast to many other countries—like Switzerland and the Netherlands (with a stronger focus on public policy) and the United States (with a stronger focus on domestic politics)—, international relations with its subfields (clusters 1, 3, 9, and 10) and the neighboring subfield of comparative politics (clusters 6 and 7 and partially 5 and 8 with a stronger focus on institutions) dominate the discipline in Germany, followed by voting and election studies with a focus on German politics (cluster 2), social policy and welfare state politics (clusters 5 and 8), and public administration (cluster 10). In the case of international relations, this may be partly due to the presence of dedicated research institutes with insulated research interests and collaboration patterns (DIE, HSFK, SWP). Yet, it is well possible that their existence is the consequence, rather than the cause, of the dominant role of international relations and comparative politics in German political science.

Second, universities and research institutes in Berlin host many of the most central researchers in German political science. Given the findings presented in this article, FU Berlin, WZB and SWP clearly dominate many of the clusters and the list of the most central scientists. This is somewhat at odds with rankings like CHE, which would place Mannheim, Konstanz, and Bremen at least at a similar position as FU Berlin in terms of research reputation. Yet, researchers from these three universities, along with researchers from Heidelberg, Munich and Frankfurt, also occupy prominent places in some of the identified research groups.

These findings imply interesting connections to existing research on bibliometrics: how exactly and to what extent does a central position in a national scientific field translate into actual ideational influence? That is, can we find ways to convert one unit on the centrality scale to a certain share of innovation in the whole network that can be attributed to the central focal node? Future research should try to establish the link between network position and actual influence more clearly (see [57] for first results). Here, we must be content with the description of network positions under the assumption that centrality in an epistemic collaboration network somehow translates into actual influence on the discipline as a whole. If such progress can be made, it may be eventually possible to replace reputation-based research rankings by network-based methods.

Moreover, future research should establish the link between the scientific growth model underlying the notions of invisible colleges and groups of collaborators of Crane [1] on the one hand and the distribution of research topics per country on the other hand. Invisible college theory suggests that the early formation of informal research clusters eventually determines what research topics get onto the agenda of a national research field. Is this process, which was posited by sociologists of science back in the 1960s and 1970s, causing German political science to focus so strongly on international relations and comparative politics? If this is true and if we are willing to assume that these processes may be applicable to political science (which is somewhat ambiguous in the literature on invisible colleges), we should find similar patterns with informal research groups focusing on public policy and administration in countries like the Netherlands and Switzerland. A comparative analysis may shed light on this cross-national variation. Moreover, it may be necessary to look at historical data on the early evolution of the discipline in order to determine whether the formation of these informal research clusters in the first place determines the current, possibly reinforced state of the network.
